# The effects of rapid weight loss on skeletal muscle in judo athletes

**DOI:** 10.1186/s12967-020-02315-x

**Published:** 2020-03-30

**Authors:** Roberto Roklicer, Nemanja Lakicevic, Valdemar Stajer, Tatjana Trivic, Antonino Bianco, Diba Mani, Zoran Milosevic, Nebojsa Maksimovic, Antonio Paoli, Patrik Drid

**Affiliations:** 1 Faculty of Sport and Physical Education, University of Novi Sad, Novi Sad, Serbia; 2 PhD Program in Health Promotion and Cognitive Sciences, University of Palermo, Palermo, Italy; 3 Sport and Exercise Sciences Research Unit, University of Palermo, Palermo, Italy; 4 Department of Applied Physiology and Kinesiology, University of Florida, Gainesville, FL USA; 5 Department of Biomedical Sciences, University of Padova, Padua, Italy

**Keywords:** Combat sports, Weight reduction, Muscle damage, Creatine kinase, Myoglobin, Aldolase

## Abstract

**Objective:**

To observe the effect of rapid weight loss (RWL) methods over 3 days on muscle damage in *judokas*.

**Methods:**

Eighteen judokas participated in this crossover study, meaning that judo athletes were subjected to exercise-only phase (4 days) and RWL phase (3 days). Subjects were tested for myoglobin, creatine kinase, aldolase, hemoglobin, and hematocrit values on seven consecutive days. These biomarkers served as indicators of acute muscle damage.

**Results:**

During the exercise-only phase, no significant changes were observed. Myoglobin (Mb) (p < 0.001), creatine kinase (CK) (p < 0.001) and aldolase (ALD) (p < 0.001) significantly increased only during the RWL phase, as well as hemoglobin (Hb) (p < 0.001) and hematocrit (Hct) (p < 0.005) values. It was detected that peak values for muscle damage biomarkers were reached on the sixth day, while Hct and Hb values were the highest on the seventh day of the study.

**Conclusion:**

Our study showed significant muscle damage induced by RWL. The prevalence of RWL use by judokas is high but firm scientific evidence is lacking in the evaluation of the current practice of it. Therefore, further knowledge must be gained to evaluate the effectiveness of RWL on performance and its impact on judokas’ wellbeing.

## Background

Active elite combat sport athletes regularly experience rigorous training regimens to succeed in competition, which often results in muscle damage (MD). Not surprisingly, MD is an inevitable aspect of any physical training at a sufficiently challenging intensity and volume [1]. In particular, combat sports are extremely demanding concerning physical training. For instance, Degoutte et al. [2] found that exercise intensity during an advanced-level judo match is equivalent to about 92% of maximal oxygen uptake (VO_2_max).

Besides maintaining excellent physical shape and fitness, elite judo athletes must maintain their optimal competitive weight for a competition, given judo is a weight-categorized sport. Therefore, judokas often engage in rapid weight loss (RWL) prior to competition to gain an advantage over their lighter opponents [3]. Namely, it has been reported that nearly 90% of judokas engage in RWL on multiple occasions per year [3]. In other combat sports, including mixed martial arts [4–6], jujitsu [5, 7], Brazilian jiu jitsu [5], boxing [8], taekwondo [5, 7, 8], kickboxing [5], and wrestling [5, 8], 60–80% of athletes have reported to engage in some form of weight-cutting. As in other combat sports, judokas often employ radical approaches to induce RWL, applying methods such as reduced fluid intake, caloric deficiency, increased physical activity, plastic suit training, heated room training, and sauna [3, 8, 9]. Combat sport athletes may even apply severe methods to lose weight, such as consuming laxatives and diuretics [7, 9] and, in some cases, forced vomiting [4, 8, 9]. Consequently, it is unsurprisingly that these extreme approaches have led to death in some cases [3]. Notably, diuretics are prohibited by the World Anti-Doping Agency (WADA) [10] and are responsible for a significant number of doping cases in combat sports [11]. A survey done in 2005 by WADA reported the highest percentage of doping cases in boxing, taekwondo and wrestling, 3.41%, 1.97% and 1.65%, respectively [11]. There is substantial evidence claiming that RWL can negatively affect athletes, compromising performance throughout the competitive season [12]. In these circumstances, combat sports athletes are at a greater risk to develop overtraining syndrome [13]. Beyond performance, many health complications have been associated with RWL, which range from acute to chronic [14]. Considering that judokas engage in RWL frequently, they can be considered *weight cyclers*, defined by the repeated loss and regain of body weight, typically by means of “yo-yo” dieting.

Although vigorous physical training is a powerful stressor to body homeostasis [15–17], it can present potentially lethal consequences when coupled with other RWL methods [12]. Isacco et al. [18] monitored athlete heart rates during judo bouts after an RWL period, finding that the mean heart rate was ~ 174 bpm and maximal was ~ 187 bpm, which represents 92% and 99% of their theoretical maximal heart rate, respectively.

From a perspective that prioritizes athlete health and wellbeing, it is important to mandate regular health screenings. Skeletal muscles play the essential role in executing movements, including the difficult movement series, as required by combat training and competition [19]. As such, the often overworked skeletal muscles of combat sport athletes will provide useful insight to an individual’s health status [20]. Recognizing the value of evaluating skeletal muscle health, we targeted the use of biochemical markers, rather than invasive and expensive methods such as muscle biopsies, to gain insight into MD in active judoka. Two reliable biomarkers recorded in our study are myoglobin (Mb) and creatine kinase (CK) [21]. Generally, CK values increase more gradually than Mb concentrations after exercise. Thus, Mb is considered a more sensitive marker of skeletal MD [22]. We also included aldolase (ALD) in our testing procedure, which, when used in conjunction with CK, may evaluate the status of muscle adaptation to training [1]. Furthermore, since judokas utilize methods to induce intentional hypohydration and therefore reduce blood plasma [23], we examined participants’ hemoglobin (Hb) to see if oxygen transportation is impaired. Hematocrit (Hct) levels were recorded to examine any changes in red blood cell count.

We hypothesized that RWL methods would cause more significant MD in comparison to everyday vigorous training sessions. We also hypothesized that RWL would impair oxygen transportation and lower red blood cell count in comparison with the weight maintenance period.

## Methods

### Participants

Eighteen males (mean body weight 85.3 ± 8.1 kg, mean age 25.3 ± 5.4 years, mean height 179 ± 6.7 cm) participated in this crossover study, meaning that judo athletes were subjected to exercise-only phase and RWL phase. All participants had to rapidly lose a minimum of 5% of their body weight during the RWL phase. Failure to successfully do so rendered the subjects to not be included in the data set. Each participant had at least 5 years of competitive experience in judo and was competing on at least a national level. Only those who had already been implementing RWL techniques over the last 2 years were selected for the study. Current literature defines RWL as a 5% weight cut achieved over a period of no more than 7 days [3, 24]. All screened participants agreed to participate in the study voluntarily, and provided signed informed consent. The study was approved by the Institutional Review Committee of the University of Novi Sad, Serbia (ref. 24/2018) and was conducted under the Declaration of Helsinki.

### Experimental design

The study was designed to observe possible alterations in MD markers during two time frames: weight-maintenance period (days 1–4) and RWL period (days 5–7). All participants used methods such as increased physical activity, plastic suit training, caloric deficit, reduced fluid intake and sauna in order to rapidly lose weight during the last 3 days. Creatine kinase (CK), myoglobin (Mb) and aldolase (ALD), blood hematocrit (Hct), and hemoglobin (Hb) levels were analyzed. Blood samples and body weight measurements were taken once in the morning on each of the 7 days of the study. Participants maintained their usual training routines during the study, which included at least one session per day. Subjects were instructed not to work out in the morning prior to blood sampling and weight measurements.

### Anthropometric profile

Participants’ body weights were measured to the nearest 0.1 kg at the same time during each day of the study (07:00 local time) using an Omron weight scale BF511 (Omron, Japan). Subjects were also instructed not to eat after 21:00 local time, thus permitting regular overnight fasting prior to measurements in the morning. Body height was measured to the nearest 0.1 cm using a Martin anthropometer (GPM, Switzerland).

### Blood sample collection

Venous blood was collected from the antecubital vein in the morning in a fasted state. All assays were done on the same day. The sampling was done by trained laboratory staff.

A 2 mL venous blood sample was collected into BD vacutainer with EDTA for each parameter (hemoglobin and hematocrit). Hematocrit concentration was analyzed using conductometry and hemoglobin concentration was analyzed using photometry (Advia 120 System, Siemens, Germany).

An 8 mL venous blood sample was collected into a serum BD vacutainer for other parameters. Creatine kinase (CK) levels were measured using a CK-NAC Reagent (Creatine Kinase, activated by N-Acetyl Cysteine) using the Dirui apparatus, recommended by the IFCC. Myoglobin (Mb) was determined by the EIA method, using a TOSOH (Tosoh, Tokyo, Japan). Aldolase levels were determined by fructose-1,6-diphosphate (F-1,6-DP) as a substrate for direct determination of aldolase. The rate of the aldolase reaction is measured by the subsequent decrease in absorbance at 340 nm as a consequence of the conversion of NADH to NAD^+^, RANDOX reagent.

### Statistical analysis

Results obtained are presented as mean values and standard deviations. The level of statistical significance was set at 5%. The analysis was conducted via repeated measures ANOVA using IBM SPSS Statistics for Windows, Version 20.0. (IBM Corp.20, Armonk, NY). The sample size was estimated using G*Power 3.1 to test for correlation and regression analyses [25], with the significance set at 0.80.

## Results

**Fig. 1:**
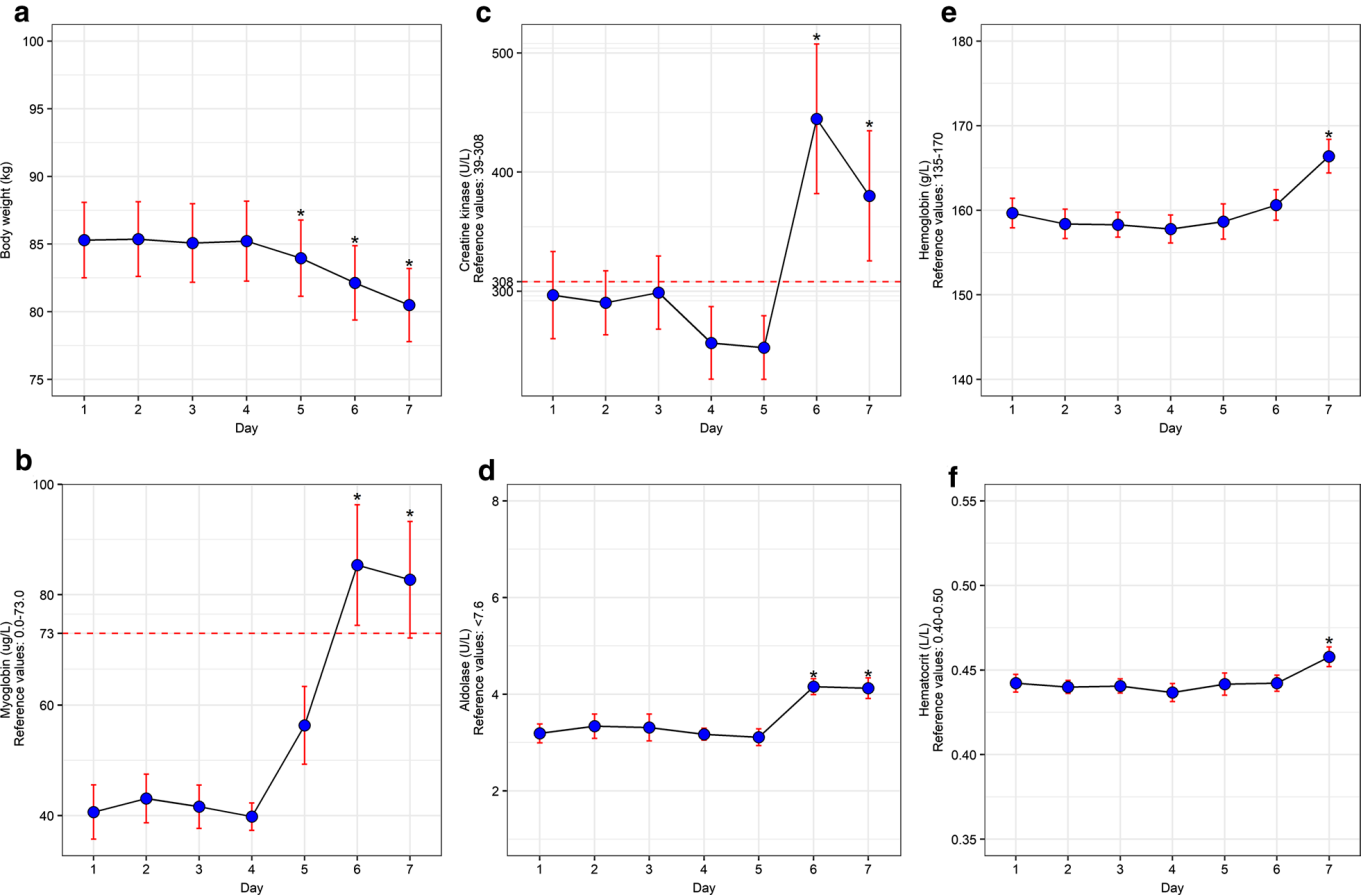
Changes in body weight and biomarkers. **a** Daily fluctuations in body weight for tested subjects. *p value (p < 0.001) represents the significance between the average weight of the exercise-only vs RWL period. F = 175.335. **b** Myoglobin levels over 7 days. *p value (p < 0.001) represents the significance between the average Mb levels of the exercise-only vs RWL period. F = 10.615. **c** Creatine kinase levels over 7 days. *p value (p < 0.001) represents the significance between the average CK levels of the exercise-only vs RWL period. F = 10.381. **d** Aldolase levels over 7 days. *p value (p < 0.001) represents the significance between the average ALD levels of the exercise-only vs RWL period. F = 11.597. **e** Hemoglobin levels over 7 days. *p value (p < 0.001) represents the significance between the average Hb levels of the exercise-only vs RWL period. F = 9.885. **f** Hematocrit levels over seven days. *p value (p < 0.005) represents the significance between the average hct levels of the exercise-only vs RWL period. F = 5.362

We observed muscle damage as a consequence of judo-specific vigorous training sessions during the first 4 days and the effects of RWL during the last 3 days of the seven-day observation period. All participants lost approximately 5% of their body weight during 3 days of RWL period (Fig. 1a). It can be noticed that the weight significantly dropped within the last 3 days of the study, from 85.22 ± 12.53 to 81.49 ± 11.43 kg, on the fourth and seventh day, respectively (F = 175.335, p < 0.001). Concurrent to weight loss, an increase in muscle damage markers (Mb, CK, ALD) was observed. Serum myoglobin levels significantly increased during the RWL period (F = 10.615, p < 0.001), with the levels exceeding the reference values on the sixth and seventh day (Fig. 1b). Creatine kinase (CK) significantly increased on the last two measurements with the values exceeding the reference values on both days (F = 10.381, p < 0.001) (Fig. 1c).

Regarding CK and Mb, a tendency to gradually reverting to stable values was present on the last day, although the values were still greater than the reference values. A significant increase in serum aldolase (ALD) was noticed among subjects (F = 11.597, p < 0.001) on the last 2 days of RWL (Fig. 1d). However, ALD levels remained within the reference range, unlike CK and Mb levels.

Blood hematocrit levels were significantly elevated on the final day of the RWL period (F = 5.362, p < 0.005) but remained within reference values (Fig. 1f). In the same manner, the significant changes were observed in blood hemoglobin (Hb) values (F = 9.885, p < 0.001) (Fig. 1e) on the last day of the study.

## Discussion

Rapid weight loss caused notable changes in MD markers over time; precisely, Mb levels during the last 3 days and CK and ALD levels during the last 2 days of the study. As the final weigh-in on the seventh day approached, body weight rapidly dropped and MD biomarkers measures increased. By following the weight maintenance period (days 1–4) of the study, it was clear that MD markers (CK, Mb and ALD) varied, but insignificantly. On the fourth day of the weight maintenance period, CK levels had declined but remained within reference values (Fig. 1). These are consistent with normal, healthy values, which range 39-308 U/L [1].

Biomarker values were more significantly elevated during the latter RWL phase. Creatine kinase (CK) levels increased rapidly on the sixth day, exceeding reference values. Although the upper limit for CK levels is typically 308 U/L, the recorded values were far beyond the reference values after a couple of days of weight loss (444.72 ± 266.13 U/L), meaning the maximal values surpassed normal values. On the last day of weight loss, CK levels dropped slightly but remained above reference values (381.96 ± 231.78 U/L).

Myoglobin (Mb) activity during the weight loss period was similar in profile to that of CK. However, on the fifth day, near the start of the RWL period, serum Mb increased significantly, although values remained within the reference range (0–73 μg/L). Day six of the study observation revealed Mb levels to reach the maximum values (85.37 ± 46.34 μg/L) and thus exceeded the reference range values. Similar to CK, Mb concentrations decreased slightly on the last day of observation, although still above reference values.

Aldolase (ALD) levels did not change significantly during the weight maintenance period, although the slight decrease could be observed across the first 4 days of observation. The last 2 days of the RWL period revealed an increase in ALD levels, with the most significant values reached on the sixth day (4.16 ± 0.70 U/L). Similar to the previously mentioned MD markers, a slight decrease appeared in ALD concentrations on the final measurement. Normal ALD levels typically range 0–7.6 U/L [1]. ALD values below 2.0 and 3.0 U/L are often observed in individuals with fructose intolerance, muscle-wasting disease, and late-stage muscular dystrophy [1]. Certainly, the protocol applied in our study was not to as extreme a level as these ailments.

Blood hemoglobin (Hb) level remained stable during exercise-only phase, but increased significantly during the RWL phase. The maximal value (166.10 g/L) was reached on the last measurement (day 7), which was still below the reference value (< 170 g/L). Similarly, blood hematocrit (Hct) values recorded at all measurements remained with a normal range, with maximal Hct concentrations on the final day of RWL (0.458 ± 0.027 L/L). Therefore, similar trends were detected both for Hb and Hct since they reached peak values on the last day of the study. Highly elevated Hct values increase blood viscosity and thus upregulate cardiac workload [26, 27]. However, Hct values obtained within our study did not go beyond reference values. Likewise, since blood plasma is lower with dehydration, making red blood cell concentration higher, Hb values are also higher temporarily. Several studies indicate that alterations of these two parameters were associated with a reduction in performance capacity [28].

Only a few previously published studies have assessed MD marker activity throughout RWL in combat sport athletes. In a study published by Isik and colleagues [29], CK levels were measured to determine the possibility of skeletal MD in junior male freestyle wrestlers who were using dehydration as a method of weight-cutting. The majority of subjects in the weight-loss group in their study (> 60%) reported having lost weight 1–7 days prior to the competition by dehydrating themselves. During the last 3 days of the study mean weight loss of weight was 4.44 ± 1.97. The difference in CK values before and after weight-loss was significant (p < 0.001). This change was similar to those observed in our study, indicating the considerable elevation of CK levels in post RWL measurements (p < 0.001).

Ozkan and Ibrahim [30] also measured alterations to CK levels as a consequence of weight reduction, reporting results similar to those of Isik et al. [29]. Ten wrestlers lost > 5% of their body weight by undergoing dehydration as their RWL method. As a result, these athletes reached peak CK values of 472.9 ± 226.3 U/L, well above reference values, indicating similar results to those found in our study. Additionally, Drid et al. [31] assessed the impact of a 5% weight cut via caloric deficiency method on creatine metabolism markers in male judokas. The results showed a significant elevation of serum creatinine in a follow-up measurement (p < 0.01). This indicates that creatine metabolites could also be affected by RWL and that, overall, skeletal MD is an apparent consequence of RWL methods.

Since the RWL period was limited to 3 days (5–7) of a total 7 days of observation in our study, the first 4 days served as a time period where MD markers were observed under stable conditions, during which athletes performed the regular daily training routine but without progressive weight loss. In fact, on average participants gained less than a kilogram (0.93 kg) of body weight on the fourth day in comparison to the first day of the study. This enabled solidified biomarker value for reference. The number of studies investigating if RWL impacted MD biomarker (CK, Mb, ALD) concentrations are rather limited. To our knowledge, this is the first study to investigate the effects of RWL in judo athletes with the exact limitation of 3 days to lose at least 5% of body weight. This is a normal number of days for weight-cutting experienced by some judokas. We adopted a crossover design in which the same group of participants was subject to measurements during the exercise-only and RWL periods. This can be seen as compensation for the lack of a control group. We were able to reach 100% adherence to our study despite a rather demanding experimental design.

Our study successfully recruited 18 volunteers, which, although adequately powered for interpretation of our results, is limiting in generalizability. Even though our athletes achieved 5% weight loss within the constraints of the protocol, 3 days might be an insufficient time frame to see a transition from short to mid-term effects. For example, MD markers had higher values on the sixth day of observation, compared to the seventh, which is unexpected. Extended days for weight loss may have expanded on the trending but non-significant finding.

Additionally, our participants were only men, which precludes us from drawing similar conclusions for women judokas, since women have different physiological and psychological responses to exercise [32–36]. Needless to say, pubescent and post-pubescent women are also more physiologically variable than men due to the menstrual cycle and hormonal changes [37, 38]. Changes that may occur due to RWL in women, especially those maintaining low weights, should be considered (i.e. symptoms of amenorrhea) [39].

Another limiting factor within our study is the absence of imaging device use such as ultrasound imaging, magnetic resonance imaging, and computed tomography to examine muscle fiber size, thickness, and shape and structural characteristics of the muscles. Due to limited budget and already intense protocol we were unable to conduct these analyses.

Further studies should elaborate how different methods may lead to different levels of muscle damage, as suggested by those studies that focused on body weight loss due to dehydration methods [29, 30]. Based on our data, we are unable to confirm the degree to which RWL caused MD and to what degree exercise contributed to it. However, a significant increase in MD markers was noted during RWL. As previously stated, intense training will likely cause MD, but, in the case of ongoing RWL, necessary nutrients are not provided to nourish the body and potentially alleviate damage in muscle tissue induced by exercise. Subjects’ Mb, CK, and ALD levels shown in our study clearly demonstrate that. Usually, vigorous training increases serum CK and Mb concentrations, but their responses can be modulated by training status [40–42], exercise modality [43, 44], and fluid intake [45, 46]. Therefore, fitness level and overall health status of an athlete undergoing RWL is a crucial factor when considering the impact of RWL on MD.

## Conclusion

Based on our findings, it appears that judokas who rapidly lost at least 5% of their body weight prior to competition are physiologically worn-out before competition. Our study provides evidence that skeletal muscles are damaged to a significant extent and that athletes who undergo rapid weight loss (RWL) and strenuous exercise would likely not be able to perform at peak levels during competition. Firm scientific evidence is lacking in the evaluation of the current practice of RWL, and must be gained to evaluate the effectiveness of the loss of weight in competitive success. To our knowledge, this is the first study that demonstrates that MD occurs among judokas even before competition. RWL induced significant MD in judokas, which can be detected by the significant increase in myoglobin, creatine kinase and aldolase values.

## Supplementary information

**Additional file 1.** Changes in body weight and biomarkers.

## Data Availability

Not applicable.

## Abbreviations

RWL: Rapid weight loss
MD: Muscle damage
CK: Creatine kinase
ALD: Aldolase
Mb: Myoglobin
Hb: Hemoglobin
Hct: Hematocrit
WADA: World Anti-Doping Agency

## References

[1] Brancaccio P, Lippi G, Maffulli N (2010). Biochemical markers of muscular damage. Clin Chem Lab Med.

[2] Degoutte F, Jouanel P, Filaire E (2003). Energy demands during a judo match and recovery. Br J Sports Med.

[3] Artioli GG, Gualano B, Franchini E, Scagliusi FB, Takesian M, Fuchs M, Lancha AH (2010). Prevalence, magnitude, and methods of rapid weight loss among Judo competitors. Med Sci Sports Exerc.

[4] Barley OR, Chapman DW, Abbiss CR (2018). Weight loss strategies in combat sports and concerning habits in mixed martial arts. Int J Sports Physiol Perform..

[5] Barley OR, Chapman DW, Abbiss CR (2019). The current state of weight-cutting in combat sports-weight-cutting in combat sports. Sports.

[6] Crighton B, Close GL, Morton JP (2016). Alarming weight cutting behaviours in mixed martial arts: a cause for concern and a call for action. Br J Sports Med.

[7] Brito CJ, Roas AF, Brito IS, Marins JC, Cordova C, Franchini E (2012). Methods of body mass reduction by combat sport athletes. Int J Sport Nutr Exerc Metab.

[8] Reale R, Slater G, Burke LM (2018). Weight management practices of Australian Olympic Combat Sport Athletes. Int J Sports Physiol Perform..

[9] Kiningham RB, Gorenflo DW (2001). Weight loss methods of high school wrestlers. Med Sci Sports Exerc.

[10] Cadwallader AB, de la Torre X, Tieri A, Botre F (2010). The abuse of diuretics as performance-enhancing drugs and masking agents in sport doping: pharmacology, toxicology and analysis. Br J Pharmacol.

[11] Halabchi F (2009). Doping in combat sports.

[12] Franchini E, Brito CJ, Artioli GG (2012). Weight loss in combat sports: physiological, psychological and performance effects. J Int Soc Sports Nutr.

[13] Franchini E, Brito CJ, Fukuda DH, Artioli GG (2014). The physiology of Judo-specific training modalities. J Strength Cond Res..

[14] Mendes SH, Tritto AC, Guilherme JPLF, Solis MY, Vieira DE, Franchini E, Lancha AH, Artioli GG (2013). Effect of rapid weight loss on performance in combat sport male athletes: does adaptation to chronic weight cycling play a role?. Br J Sports Med.

[15] van Praag H, Fleshner M, Schwartz MW, Mattson MP (2014). Exercise, energy intake, glucose homeostasis, and the brain. J Neurosci.

[16] Davies KJA (2018). Cardiovascular adaptive homeostasis in exercise. Front Physiol..

[17] Mendez-Villanueva A, Fernandez-Fernandez J, Bishop D (2007). Exercise-induced homeostatic perturbations provoked by singles tennis match play with reference to development of fatigue. Br J Sports Med.

[18] Isacco L, Degoutte F, Ennequin G, Pereira B, Thivel D, Filaire E. Rapid weight loss influences the physical, psychological and biological responses during a simulated competition in national judo athletes. *Eur J Sport Sci.* 2019;1-12. 10.1080/17461391.2019.1657503 31418331

[19] Reale R, Burke LM, Cox GR, Slater G (2019). Body composition of elite Olympic combat sport athletes. Eur J Sport Sci..

[20] Lindstedt SL (2016). Skeletal muscle tissue in movement and health: positives and negatives. The J Exp Biol..

[21] Yamamoto LM, Judelson DA, Farrell MJ, Lee EC, Armstrong LE, Casa DJ, Kraemer WJ, Volek JS, Maresh CM (2008). Effects of hydration state and resistance exercise on markers of muscle damage. J Strength Cond Res..

[22] Driessen-Kletter MF, Amelink GJ, Bär PR, van Gijn J (1990). Myoglobin is a sensitive marker of increased muscle membrane vulnerability. J Neurol.

[23] Reljic D, Hassler E, Jost J, Friedmann-Bette B (2013). Rapid weight loss and the body fluid balance and hemoglobin mass of elite amateur boxers. J Athl Train..

[24] Berkovich BE, Eliakim A, Nemet D, Stark AH, Sinai T (2016). Rapid weight loss among adolescents participating in competitive judo. Int J Sport Nutr Exerc Metab.

[25] Faul F, Erdfelder E, Buchner A, Lang AG (2009). Statistical power analyses using G*Power 3.1: tests for correlation and regression analyses. Behav Res Methods.

[26] El-Sayed MS, Ali N, Ali ZES (2005). Haemorheology in exercise and training. Sports Med.

[27] Böning D, Maassen N, Pries A (2011). The hematocrit paradox–how does blood doping really work?. Int J Sports Med.

[28] Connes P, Simmonds MJ, Brun JF, Baskurt OK (2013). Exercise hemorheology: classical data, recent findings and unresolved issues. Clin Hemorheol Microcirc..

[29] Isik O, Yildirim I, Ersoz Y, Koca HB, Dogan I, Ulutas E (2018). Monitoring of pre-competition dehydration-induced skeletal muscle damage and inflammation levels among elite wrestlers. J Back Musculoskelet Rehabil..

[30] Ozkan I, Ibrahim CH (2016). Dehydration, skeletal muscle damage and inflammation before the competitions among the elite wrestlers. J Phys Ther Sci..

[31] Drid P, Krstulović S, Erceg M, Trivic T, Stojanovic M, Ostojic S (2019). The effect of rapid weight loss on body composition and circulating markers of creatine metabolism in judokas. Kinesiology..

[32] Granito VJ (2002). Psychological Response to Athletic Injury: gender Differences. J Sport Behav..

[33] Hunter SK (2014). Sex differences in human fatigability: mechanisms and insight to physiological responses. Acta Physiol.

[34] Hunter SK (2016). Sex differences in fatigability of dynamic contractions. Exp Physiol.

[35] Reider B (2012). Sex in sports medicine. Am J Sports Med.

[36] Tarnopolsky MA (2008). Sex differences in exercise metabolism and the role of 17-beta estradiol. Med Sci Sports Exerc.

[37] Bruinvels G, Burden RJ, McGregor AJ, Ackerman KE, Dooley M, Richards T, Pedlar C (2017). Sport, exercise and the menstrual cycle: where is the research?. Br J Sports Med.

[38] Oosthuyse T, Bosch AN (2010). The effect of the menstrual cycle on exercise metabolism: implications for exercise performance in eumenorrhoeic women. Sports Med.

[39] Prouteau S, Ducher G, Serbescu C, Benhamou L, Courteix D (2007). Gender differences in response to weight cycling in elite judoists. Biol Sport..

[40] Clarkson PM, Hubal MJ (2002). Exercise-induced muscle damage in humans. Am J Phys Med Rehabil.

[41] Clarkson PM, Tremblay I (1988). Exercise-induced muscle damage, repair, and adaptation in humans. J Appl Physiol.

[42] Koutedakis Y, Raafat A, Sharp NC, Rosmarin MN, Beard MJ, Robbins SW (1993). Serum enzyme activities in individuals with different levels of physical fitness. J Sports Med Phys Fitness.

[43] Clarkson PM, Kroll W, Graves J, Record WA (1982). The relationship of serum creatine kinase, fiber type, and isometric exercise. Int J Sports Med.

[44] Volek JS, Kraemer WJ, Rubin MR, Gomez AL, Ratamess NA, Gaynor P (2002). L-Carnitine L-tartrate supplementation favorably affects markers of recovery from exercise stress. Am J Physiol Endocrinol Metab.

[45] Clarkson PM, Kearns AK, Rouzier P, Rubin R, Thompson PD (2006). Serum creatine kinase levels and renal function measures in exertional muscle damage. Med Sci Sports Exerc.

[46] Seifert JG, Kipp RW, Amann M, Gazal O (2005). Muscle damage, fluid ingestion, and energy supplementation during recreational alpine skiing. Int J Sport Nutr Exerc Metab.

