# Supplementary figures and images for: The effects of rapid weight loss on skeletal muscle in judo athletes
Source: J Transl Med. 2020 Mar 30;18:142. doi: 10.1186/s12967-020-02315-x (PMC7106841; doi:10.1186/s12967-020-02315-x)

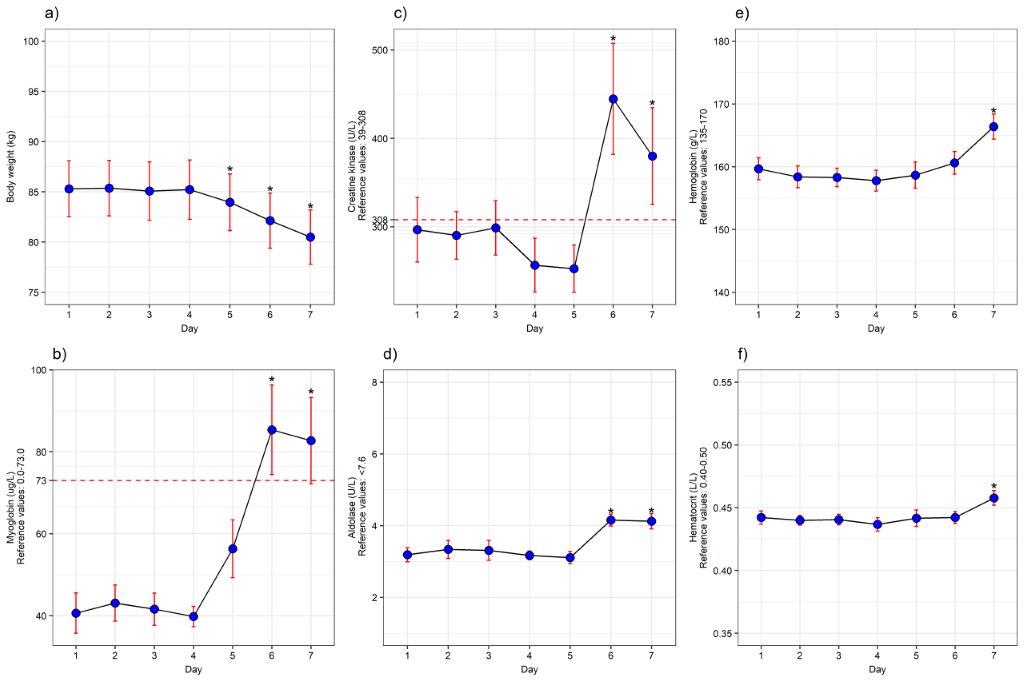

Supplement: Supplementary file 1 — Additional file 1. Changes in body weight and biomarkers. [file 12967_2020_2315_MOESM1_ESM.jpg]
